# School Burnout Inventory: Latent Profile and Item Response Theory Analyses in Undergraduate Samples

**DOI:** 10.3389/fpsyg.2020.00188

**Published:** 2020-03-06

**Authors:** Ross W. May, Peter M. Rivera, Ronald D. Rogge, Frank D. Fincham

**Affiliations:** ^1^Family Institute, Florida State University, Tallahassee, FL, United States; ^2^School of Psychology, Family, and Community, Seattle Pacific University, Seattle, WA, United States; ^3^Department of Clinical and Social Sciences in Psychology, University of Rochester, Rochester, NY, United States

**Keywords:** item response theory, latent profile analysis, person-oriented approach, repeated measures latent profile analysis, School Burnout Inventory

## Abstract

The current research reports both latent profile (person-oriented) and item response theory (IRT) analyses of the School Burnout Inventory (SBI) in United States undergraduate samples. Study 1 (*n* = 1,007) comprises a latent profile analysis (LPA) that identified four mutually exclusive subgroups based on patterns of school burnout responses. Covariate analyses of grade point average and negative affect suggested that school burnout profiles function similarly to variable-oriented approaches. Study 2 (*n* = 544) explored longitudinal patterns of school burnout among college students via use of a repeated measures LPA. Findings suggested that the profiles identified reflect a relatively stable school burnout trajectory over time. Covariate analysis of sleep quality and academic engagement demonstrated differences across profiles, but the patterns were similar to variable-oriented statistical approaches. Study 3 (*n* = 2,364) utilized an IRT analysis of the SBI to identify a short, efficient measure. Item information curves and graded response model item discrimination parameters identified a 4-item SBI scale (SBI-4) that offered reasonably high levels of information for assessing school burnout in comparison to the original nine-item SBI. Implications and future research are identified.

## Introduction

The attainment of a baccalaureate degree offers a considerable advantage in job placement over those who have not acquired an undergraduate education (U.S. Department of Commerce, 2016). Not surprisingly, approximately 69.2% of high school graduates go on to enroll in college programs (U.S. Department of Commerce, 2016). Undergraduate enrollment is disproportionally female, with about 11.5 million females enrolled in the fall of 2017 as compared to 8.9 million males (U.S. Department of Education, 2017). Reflecting the importance of higher education, the number of American undergraduate students is increasing. Enrollment of traditional college-aged students (18- to 24-year-olds) is up more than 5.1 million in 2017 (20.4 million) from that of 2000 (U.S. Department of Education, 2017). Given the prevalence of college education and its resulting employment advantages, investigations into factors that may jeopardize retention in United States colleges are warranted. One such emerging factor is school burnout.

Burnout is a well-researched, multidimensional, affective response to occupational stress. Although burnout has traditionally been investigated within the workplace, it is becoming more common to extend burnout investigations into academic populations (Salmela-Aro et al., 2008, 2009; Parker and Salmela-Aro, 2011). Within an educational context, *school* burnout has been characterized by chronic exhaustion from school-related work, cynicism toward the meaning of school, and feelings of inadequacy toward school-related accomplishments (Salmela-Aro et al., 2008, 2009). A host of negative conditions have been associated with school burnout, including suboptimal physiological functioning (May et al., 2014a, b, 2016) and diminished cognitive and academic performance (Salmela-Aro et al., 2008, 2009; May et al., 2015).

Although school burnout is typically studied using a variable-oriented approach, valuable contributions stemming from a person-oriented approach have improved the school burnout literature. Contrary to research treating school burnout symptoms as the unit of analysis, person-oriented analyses focus on groups of individuals as the unit of analysis. As thoroughly discussed in Mäkikangas and Kinnunen (2016), person- and variable-oriented statistical approaches diverge both theoretically and methodologically. These approaches differ statistically in that person-oriented analyses examine intra-individual variation in school burnout symptomology (e.g., class or cluster analyses), whereas variable-oriented analyses examine inter-individual variation (e.g., linear association between variables through either correlational or mean level analyses). Person-oriented statistical analyses assume that there is heterogeneity in burnout symptoms in the population (Laursen and Hoff, 2006).

Typically, person-oriented analyses begin with an unknown number of classes or clusters and proceed with the development of different class solutions. Class solutions are then compared and evaluated on the basis of statistical and theoretical criteria (Bergman et al., 2003; Mäkikangas and Kinnunen, 2016). The advantage of a person-oriented approach is its ability to identify types or classes of symptomology and the trajectory of development and change in symptomology. Types or classes are not identified by any predefined scores or cutoff values and, in the context of school burnout, may help to provide a unique perspective on how individuals are similar (or different) from each other in burnout symptomology. Thus, person-oriented statistical approaches help identify how many groups of individuals are needed to largely describe between-person differences (either longitudinally or cross-sectionally), assuming that they are drawn from several subpopulations with between-person variability.

The systematic review conducted by Mäkikangas and Kinnunen (2016) identified only 24 publications (out of 470) that reported findings pertaining to person-oriented approaches to modeling burnout between the years 2004 and 2015. Their findings reveal that person-oriented analyses of burnout types and trajectories largely parallel burnout findings from variable-oriented approaches: burnout typically develops with the appearance of exhaustion and cynicism occurring simultaneously and then the development of reduced professional efficacy followed by symptoms that are largely stable over time. However, person-oriented analyses provided unique information regarding the identification of atypical burnout types and developmental trajectories (e.g., cynicism occurring alone or together with reduced professional efficacy).

Importantly, only 4 of the 24 studies identified by Mäkikangas and Kinnunen (2016) pertained to school burnout (the remainder focused on occupational burnout). Furthermore, none of the four publications utilized American samples (one publication utilized Korean students, one utilized Chinese students, and two utilized Finnish students; Lee et al., 2010; Zhang et al., 2013; Salmela-Aro and Upadaya, 2014; Tuominen-Soini and Salmela-Aro, 2014, respectively). In addition, only one of those publications reported on a collegiate sample (see Tuominen-Soini and Salmela-Aro, 2014, modeling burnout and engagement profiles, although see the more recent work of Salmela-Aro and Read, 2017). Differences in tuition price, enrollment rates, university degree curriculum structure, and typical class size are just a few of many factors (for additional factors, see Taylor, 2012; Chang and Wang, 2017; Chatlani, 2018) that may lead to differential student stress across various countries and across different educational settings within countries (e.g., university, technical college, community college).

Person-oriented analyses provide a worthwhile supplement to variable-oriented approaches to examining school burnout. However, given their limited use in the school burnout literature and the lack of representation of American collegiate samples in the person-oriented literature, the current research reports findings of latent profile analyses (LPA) of school burnout in American collegiate samples. Study 1 reports on a LPA examining school burnout in United States undergraduates. Study 2 then explores longitudinal patterns of school burnout among United States college students using repeated measures LPA (RMLPA). Finally, supplementing the person-oriented approaches of Studies 1–3 utilizes an item response theory (IRT) analysis to identify an efficient, psychometrically optimized 4-item School Burnout Inventory (SBI) scale (SBI-4). All three studies focus on the measurement of school burnout using the SBI (Salmela-Aro et al., 2009).

## Study 1

Person-oriented approaches to burnout complement traditional variable-oriented approaches by helping to reveal intra-individual heterogeneity within the burnout syndrome. This allows for the identification of types or patterns of burnout symptomology within individuals. Study 1 explores school burnout via a person-oriented approach, specifically LPA, using the SBI (Salmela-Aro et al., 2009). LPA is a latent variable modeling technique that classifies individuals into mutually exclusive subgroups based on patterns of responses to either continuous or a combination of categorical and continuous observations (Roesch et al., 2010). Extending LPA, this study also examines the associations between school burnout profile membership, related negative affect symptomology (depression and anxiety symptoms), and an indicator of academic achievement (grade point average, GPA). Prior variable-oriented research has documented relationships between the aforementioned constructs indicating that even when controlling for affective symptoms, increased school burnout predicts lower GPA (May et al., 2015).

### Analytical Approach

To explore heterogeneity within school burnout among United States college students, a LPA was conducted using Mplus (Version 7.4). The LPA focused on participants’ responses to the nine items from the SBI (Salmela-Aro et al., 2009). Independent models were estimated in an iterative manner that began with a one-profile solution, and profiles were added until the best-fitting model was identified. For each model estimated, random parameter start values were used to verify the replication of the log-likelihood value and to ensure that model convergence was due to a global rather than local maximum (Hipp and Bauer, 2006). Any missing data were handled using full-information-maximum-likelihood.

The final model was chosen based on a number of statistical indicators, model parameters, and interpretative meaning of the final solution. Specifically, absolute model fit was assessed by the following fit criterion: Akaike information criterion (AIC), Bayesian information criterion (BIC), and sample size adjusted BIC (A-BIC). Lower AIC, BIC, and A-BIC indicate better model data fit (Roesch et al., 2010). Bootstrap likelihood ratio tests (BLRTs) also informed this process, and provided a *p-*value that, when significant, indicated a model with *k* number of profiles to be preferred over a model with *k* - 1 profiles. Two parameters estimated in latent profile models also informed the selection of the final model: latent class probabilities (i.e., likelihood of belonging to a particular class) and conditional response means (i.e., mean of an item within a particular profile). Higher latent class probabilities and distinguished conditional response means are preferred. Entropy was considered in this process, which is an indicator of profile enumeration and ranges from 0 to 1, where closer to 1 indicates how well profiles have been distinguished (Roesch et al., 2010). Models that contained profiles with less than 5% of the sample were considered to be less parsimonious and less likely to replicate in future samples, and were therefore considered undesirable solutions independent of the other fit indices.

A three-step approach was used to examine the associations between profile membership and GPA and depressive and anxiety symptomology. This approach is preferred to the commonly used classify–analyze approach when determining latent profile differences on auxiliary variables because it makes use of the latent measurement model by accounting for measurement error and because of its advanced performance in examining associations between latent classes and external variables (Bolck et al., 2004; Vermunt, 2010; Asparouhov and Muthén, 2014). Following identification of the best-fitting latent profile model, the next steps involved using the posterior distribution to create an indicator of most likely latent class membership before estimating the final model with auxiliary variables (i.e., indicators of emerging adult GPA, anxiety, and depression), while accounting for the measurement error associated with latent profile classification (Asparouhov and Muthén, 2014). This process utilized Wald chi-square significance tests to determine mean differences for each outcome across identified profiles.

### Method

#### Participants

Participants were 1,007 undergraduate students (67% females, *M*_*age*_ = 20.06 years, SD = 1.26). Students who completed at least one full academic semester were eligible for study participation. Sample demographics include: 66% Caucasian, 16% African American, 1.5% Asian, 8% Hispanic, and 8.5% endorsing either biracial or non-disclosed ethnicity, with 14% freshmen, 26% sophomores, 35% juniors, and 25% seniors.

#### Measures

##### School burnout

School burnout was measured using the SBI (Salmela-Aro et al., 2009). The SBI consists of nine items measuring three first-order factors of school burnout: (a) exhaustion at school (four items), (b) cynicism toward the meaning of school (three items), and (c) sense of inadequacy at school (two items). Summed scores from the first-order factors comprise a second-order overall school burnout score. Higher composite scores indicate higher burnout. Participants responded to items (e.g., “I feel overwhelmed by my schoolwork” as an example exhaustion item, “I feel a lack motivation in schoolwork and often think of giving up” as an example cynicism item, and “I often have feelings of inadequacy in my schoolwork” as an example inadequacy item) on a scale from 1 (*completely disagree*) to 6 (*completely agree*). Internal consistency for the present sample was α = 0.89.

##### Depressive symptoms

Depressive symptomology was assessed via the 10-item Center for Epidemiological Studies Depression Scale (CES-D; Radloff, 1977; Santor and Coyne, 1997). The CES-D has been widely used as a measure of depressive symptoms in non-clinical samples. It asks participants to respond to a list of ways they may have felt or behaved during the previous week. Sample items include “I could not ‘get going”’ and “I felt hopeful about the future.” Participants responded 0 (*rarely*) to 3 (*most/all of the time*) on items, such as feelings of loneliness, hopelessness, and restless sleep. Some items were reverse-coded, such that higher responses indicate more depressive symptoms. Internal consistency for the present sample was α = 0.89.

##### Anxiety symptoms

Anxiety was measured using the 20-item State–Trait Anxiety Inventory (STAI; Spielberger et al., 1970). Participants were asked to respond to anxiety items such as “upset,” “calm,” “secure,” “at ease,” and “nervous.” Responses were scored on a four-point Likert scale from 1 (*not at all*) to 4 (*very much so*). Half of the items were reverse-coded so that higher scores indicated greater anxiety. Items were summed to create a composite anxiety score with a possible range of 20–80. Internal consistency for the sample was α = 0.90.

##### Academic achievement

Academic achievement was assessed through self-reported cumulative GPA ranging from 1.50 to 4.00.

#### Procedure

Data from all eligible participants was collected via online survey questionnaires. Questionnaires contained demographic questions and the measurement scales described. All participants were recruited from undergraduate classrooms at a large southern university in the United States as an option for voluntary class credit. Extra credit was generally less than 1% of the final grade. Data were collected in the middle (weeks 3–9) of the spring academic semester. All participants gave their written consent prior to study participation, and approval was obtained from the institutional review board before any data were collected.

### Results and Discussion

#### Selection of Optimal Latent Profile Solution

Statistical indicators used to determine the final profile solution are presented in Table 1. The analysis of the nine school burnout items suggested that a four-profile solution fit the data best. AIC, BIC, and A-BIC all decreased for every solution over one, while BLRT indicated that a five-profile solution was preferred over a four-profile solution. When comparing four- and five-profile models, however, entropy was stronger for a four-profile solution, and latent class probabilities were consistently higher for a four-profile solution. Moreover, the latent class probabilities of subgroup membership assignment were strong for the four-class solution: 91% for profile 1, 87% for profile 2, 93% for profile 3, and 93% for profile 4. For these reasons, we considered the four-profile solution to be the best-fitting model.

**TABLE 1:** Comparison of latent profile models (*N* = 1,007).

Model	AIC	BIC	A-BIC	Entropy	BLRT	Profile: *n*	LCP
1 profile	31,039.12	31,127.59	31,070.42	n/a	n/a	1. 1,007	
2 profiles	28,688.57	28,826.19	28,737.26	0.83	*p* < 0.001	1. 446	0.94
						2. 561	0.95
3 profiles	27,776.51	27,963.27	27,842.58	0.87	*p* < 0.001	1. 311	0.95
						2. 157	0.93
						3. 539	0.94
**4 profiles**	**27,524.96**	**27,760.86**	**27,608.41**	**0.84**	***p* < 0.01**	**1. 128**	**0.91**
						**2. 254**	**0.87**
						**3. 487**	**0.93**
						**4. 138**	**0.93**
5 profiles	27,352.23	27,637.28	27,453.07	0.79	*p* < 0.001	1. 157	0.91
						2. 163	0.85
						3. 320	0.84
						4. 275	0.84
						5. 92	0.93

*AIC, Akaike information criterion; BIC, Bayesian information criterion; A-BIC, sample size adjusted BIC; BLRT, bootstrap likelihood ratio test; n/a, not available; *n*, number of participants; LCP, latent class probability. Bold font indicates selected model.*

#### Description of Identified Profiles

**FIGURE 1:**
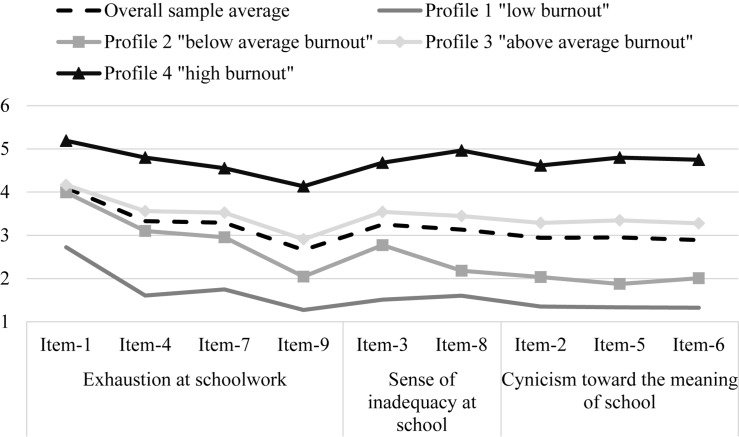
Conditional response means for a four-profile model.

The conditional response means for each indicator of school burnout were used to interpret each subgroup and are presented in Figure 1. Profile 1 participants (approximately 13% of the overall sample) reported the lowest levels of school burnout across all indicators and were labeled *low burnout*. Participants in profile 2 (approximately 25% of the overall sample) reported higher levels of burnout than their counterparts in profile 1 but lower-than-average responses overall. As such, this profile was labeled *below-average burnout*, reflecting their close yet lower-than-average responses across all indicators. Profile 3 was the largest profile, including close to half of the overall sample (48%). These participants reported above-average scores of school burnout across all nine indicators; therefore, profile 3 was labeled *above-average burnout*. Lastly, profile 4 (approximately 14% of the overall sample) was labeled *high burnout* because it comprised those with the highest reported levels of school burnout across all indicators.

#### Differences in GPA as a Function of Profile Membership

**FIGURE 2:**
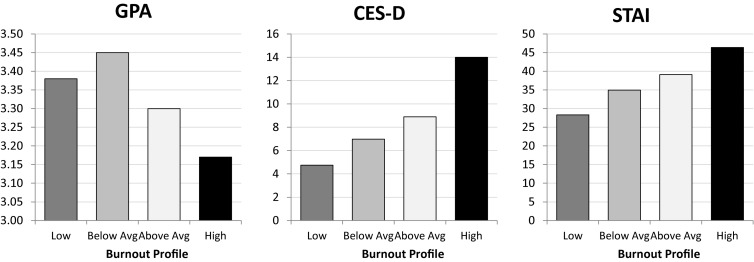
Study 1 differences on grade point average (GPA), depressive symptoms [Center for Epidemiological Studies Depression Scale (CES-D)], and anxiety [State–Trait Anxiety Inventory (STAI)] symptoms across the four burnout profiles.

Results from Wald chi-square significance tests determining whether differences existed across profile mean scores of GPA are presented in Figure 2, with Table 2 showing all *post hoc* comparisons. Findings showed that levels of GPA varied across profiles. As expected, participants comprising the *low burnout* profile reported the second highest GPA (*M* = 3.38), which was significantly higher than that of those classified into the *high burnout* profile. Those in the *below-average burnout* profile recorded the highest GPA (*M* = 3.45), which was significantly higher than that of those in the *above-average burnout* (*M* = 3.30) and *high burnout* (*M* = 3.17) profiles. Notably, participants in the *high burnout* profile reported the lowest GPA, which was statistically significantly different from each of the other profiles.

**TABLE 2:** Profile differences in grade point average, depression, and anxiety (*N* = 1,007).

Outcome *M* (SE)	Profile 1: “low burnout”	Profile 2: “below-average burnout”	Profile 3: “above-average burnout”	Profile 4: “high burnout”
GPA	3.38(0.04)^a^	3.45(0.03)^b,c^	3.30(0.02)^b,d^	3.17(0.04)^a,c,d^
Depressive symptoms	4.75(0.28)^a,b,c^	6.98(0.68)^a,d^	8.90(0.45)^b,e^	14.00(0.53)^c,d,e^
Anxiety symptoms	28.32(0.79)^a,b,c^	34.96(0.73)^a,d,e^	39.13(0.52)^b,d,f^	46.39(1.00)^c,e,f^

**M*, mean; SE, standard error, GPA, grade point average. ^*a, b, c, d*^, matching subscripts denote significant *post hoc* differences.*

#### Differences in Depressive Symptomology as a Function of Profile Membership

Findings determining profile differences in depressive symptomology are reported in Figure 2, with *post hoc* comparisons in Table 2. Participants comprising the *low burnout* profile reported the lowest levels of depressive symptomology (*M* = 4.75), followed by those in the *below-average burnout* profile (*M* = 6.98) and *above-average burnout* profile (*M* = 8.90), with individuals in the *high burnout* profile reporting the highest levels of depressive symptomology (*M* = 14.00). All profile comparisons yielded statistically significant differences except for that between participants in the *below-average burnout* and *above-average burnout* profiles.

#### Differences in Anxiety Symptomology as a Function of Profile Membership

Figure 2 also shows results from the Wald chi-square significance tests for profile differences on reported levels of anxiety symptomology, with *post hoc* comparisons in Table 2. Levels of anxiety symptomology significantly varied across all profiles. Participants in the *low burnout* profile reported the lowest levels of anxiety symptomology (*M* = 28.32), followed by those in the *below-average burnout* profile (*M* = 34.96) and then the *above-average burnout* profile (*M* = 39.13), with individuals in the *high burnout* profile reporting the highest levels of anxiety symptomology (*M* = 46.39).

These findings are novel as they reflect the first person-oriented approach to understanding intra-individual heterogeneity in burnout symptomology in a United States collegiate sample. Four unique categories of school burnout symptomology were established with a four-class profile solution: categories labeled *low burnout* (13% of the overall sample), *below-average burnout* (25% of the overall sample), *above-average burnout* (48% of the overall sample), and *high burnout* (14% of the overall sample). Similarly, Ahola et al. (2014), albeit in an older sample of employed individuals, identified a similar clustering of burnout and depressive symptoms, identifying a three-factor solution of “low level of symptoms” (low level of burnout and depressive symptoms), “intermediate level of symptoms” (intermediate levels of burnout and depressive symptoms), and “high level of symptoms” (high levels of burnout and depressive symptoms).

Interestingly, covariate analyses suggest that profiles function similarly to a variable-oriented approach. This is evidenced by a reduction in GPA and increase in affective symptoms corresponding to the increase in burnout symptomology found within the established profiles. However, as this is the first LPA using the SBI in an American collegiate sample, supplemental profile analyses would be advantageous. Toward this objective, Study 2 provides an investigation of developmental trajectories of school burnout profiles.

## Study 2

To date, consensus regarding a school burnout trajectory remains elusive. Earlier work regarding development change in burnout in European students seemed to suggest that school burnout is surprisingly consistent over time (Parker and Salmela-Aro, 2011; Salmela-Aro and Upadaya, 2014). More recent work in latent profiles, however, has suggested that while a stable, zero-slope burnout trajectory may be found within a majority of students sampled (Salmela-Aro and Upadaya, 2014), substantial trajectory heterogeneity can be identified within subclasses (see Salmela-Aro and Read, 2017). Although informative, such school burnout trajectory analyses have not yet been examined within any United States student population. Thus, to improve upon the cross-sectional limitation of Study 1 and begin to develop an idea of the developmental trajectory of school burnout over time in the United States, this study explores longitudinal patterns in school burnout profiles across three academic semesters.

Additionally, associations between school burnout profiles and student’ levels of sleep quality and academic engagement were examined. Both diminished sleep quality and poor job engagement are well-documented correlates of increased occupational burnout (for a review of sleep and occupational burnout findings, see Akerstedt et al., 2017; for a review of job engagement and occupational burnout findings, see Leon et al., 2015 or Taris et al., 2017). However, considerably less is known of these relationships in regard to school burnout, and then even less in non-European academic populations, demonstrating a need for research regarding sleep and engagement relationships with school burnout in American collegiate samples.

Sleep is a vital component of health and has been shown to be important in occupational burnout (Alvarez and Ayas, 2007; Akerstedt et al., 2017). However, research linking sleep and school burnout is largely absent in primary to post-secondary student populations. What little research there is primarily focuses on sleep–burnout relationships in medical students (for a review, see Dyrbye et al., 2006). As an example of a typical finding, a survey study of medical students in India found that poorer sleep as measured with the Pittsburgh Sleep Quality Index (PSQI) corresponded to increased subscale school burnout scores (e.g., burnout and disengagement) using the Oldenburg Burnout Inventory (Shad et al., 2015).

Engagement is important within the burnout literature as it represents motivational, cognitive, and behavioral components that may prevent burnout’s occurrence and continued development (although the uniqueness of burnout and engagement as constructs is admittedly controversial; see Taris et al., 2017). In higher education, academic engagement is conceptualized as a three-dimensional construct, comprising energy for, dedication toward, and absorption in schoolwork. Specifically, academic engagement is thought of as a positive approach to schoolwork (energy), dedication to a positive attitude while perceiving schoolwork as meaningful (dedication), and absorption in concentration on schoolwork (absorption; Salmela-Aro and Upadaya, 2014, p. 60; see also Schaufeli et al., 2002a). Attempts to link school burnout and school engagement have been limited and have primarily involved European samples, with no findings pertaining to United States students (to the best of our knowledge). However, the research that does exist indicates that decreased school engagement corresponds to a variety of negative outcomes, including increased depression and increased school burnout (Salmela-Aro et al., 2009). Demonstrating similar relationships, a longitudinal profile approach by Salmela-Aro and Read (2017) evaluating school burnout and academic engagement indicated that as academic engagement decreases, school burnout increases over time.

Therefore, given the importance of sleep and engagement and their absence in the school burnout literature, we examined their relationships with profiles of school burnout. As suggested by prior research on medical students, we expect profiles with increased school burnout symptomology to align with poorer sleep quality. Furthermore, as suggested by the findings in Salmela-Aro and Read’s (2017) study, we expected school burnout and academic engagement to be inversely related so that increased engagement corresponds to lower school burnout.

### Analytical Approach

To explore longitudinal patterns of school burnout among college students, an RMLPA was conducted in Mplus (Version 7.4). RMLPA is an application of LPA to repeated measures that aims to identify a categorical latent variable that underlies the heterogeneity in a population’s responses to questions over time (Collins and Lanza, 2010). We again focused on participants’ responses to the nine items from the SBI obtained across three time points. Independent RMLCA models were estimated in the same iterative manner that the latent profile models were estimated in the previous study, and the same statistical indicators (i.e., AIC, BIC, A-BIC, BLRT, and entropy) and model parameters (i.e., latent class probabilities and conditional response means) were used to select the optimal fitting model. Once the final model was identified, a three-step approach as conducted in Study 1 (Asparouhov and Muthén, 2014) was used to examine the associations between profile membership and student’ levels of sleep and academic engagement.

### Method

#### Participants

Participants were undergraduate students recruited from undergraduate courses at a large southern university in the United States who had completed at least one full academic semester and were enrolled in classes during the entire duration of the study. Students who completed three consecutive academic semesters (thus providing the three data waves necessary for the repeated measures analysis) were included in analyses. Students who graduated during the course of the study were eliminated from analyses. Studies 1 and 2 samples were independent in that no student was included in both analyses.

An initial sample of 989 students were contacted during the first semester (wave) of data collection. Of those students, 422 students (43% of the initial sample) graduated during the course of the study and were thus not eligible for study analysis. Of the remaining 585 students, 544 (67% females, *M*_*age*_ = 19.00 years, SD = 1.01) completed three consecutive academic semesters and were included in the analyses, resulting in 7% study attrition of eligible participants. Of study eligible participants, 66% reported as Caucasian, 16% African American, 1.5% Asian, 8% Hispanic, and 8.5% either biracial or non-disclosed ethnicity. At the time of initial data collection, 26% of students reported as freshmen, 38% sophomores, and 36% juniors. Study incentive was provided (as per instructor discretion) in the form of extra credit that did not surpass 1% of the final grade.

#### Measures

##### School burnout

School burnout was measured using the SBI as in Study 1. Reliability for the present sample was α = 0.89.

##### Sleep disturbance

Sleep was measured using the PSQI (Buysse et al., 1989). The PSQI contains 19 self-rated items that include seven clinically derived subscales measuring: subjective sleep quality, sleep latency, sleep duration, habitual sleep efficiency, sleep disturbances, use of sleeping medications, and daytime dysfunction (excludes rating provided by bed partner). Responses range from open-ended format to Likert-type scales. Subscales are comprised of unique algorithms. For example, subjective sleep quality is measured via participants’ response to the item, “During the past month, how would you rate your sleep quality overall?” Responses range from 0 (*very good*) to 4 (*very bad*), with higher scores indicating poorer sleep quality. In contrast, sleep efficiency is computed as a percentile ranging from 0 to 100, with higher scores indicating improved sleep efficiency and lower sleep problems. The algorithm for sleep efficiency exists as the value of the item “During the past month, how many hours of actual sleep did you get at night?” divided by the difference in hours between the two items “During the past month, when have you usually gone to bed?” and “During the past month, when have you usually gotten up in the morning?” multiplied by 100. Combined subscale scores are then used to derive the construct of total sleep disturbance. To achieve unilateral direction indicating higher total sleep disturbance, the subscales of sleep efficiency and duration of sleep were reverse-coded to match algorithms for sleep disturbance, sleep latency, day dysfunction due to sleepiness, overall sleep quality, and use of sleep medication. Higher scores in each subscale independently and summed together indicate poorer sleep. All PSQI sample subscale reliabilities were greater than α = 0.85.

##### Academic engagement

A 17-item measure of academic engagement developed by Schaufeli et al. (2002b) was used. The measure reflects the three underlying dimensions of engagement: energy (six items, e.g., “When I get up in the morning, I feel like going to class”); dedication (five items, e.g., “I am enthusiastic about my studies”); and absorption (six items, e.g., “When I am studying, I forget everything else around me”). All items are scored on a seven-point frequency rating scale ranging from 1 (*never*) to 7 (*always*). Items were summed to create a global composite score, with higher scores indicating greater academic engagement. Reliability for the present sample was α = 0.88.

#### Procedure

Data collection from all eligible participants was completed via online survey questionnaires over three consecutive academic semesters. Questionnaires contained demographic questions and the measurement scales described. All participants were recruited from undergraduate classrooms as an option for voluntary class credit. Extra credit was generally less than 1% of the final grade. Data were collected between weeks 3 and 5 of the academic semesters. All participants gave their consent prior to study participation, and approval was obtained from the institutional review board before any data were collected. Recruitment and data collection procedures were identical across semesters. Sleep and academic engagement measurement responses, however, were only collected during the last wave of assessments.

### Results and Discussion

#### Selection of Optimal Latent Profile Solution

Analyses of the nine school burnout items suggested a six-profile solution as the best-fitting model. As seen in Table 3, AIC, BIC, and A-BIC were lowest for a six-profile solution, and BLRT indicated a six-profile over a five-profile solution. It is worth noting that across all models, classification certainty (entropy values >0.90) and latent class probabilities (0.93–0.98) were particularly high. However, due to not having enough participants to represent each profile in the six-profile solution (i.e., profiles smaller than 5% of the overall sample), we decided to retain a five-profile model.

**TABLE 3:** Comparison of latent profile models in study 2 (*N* = 544).

Class solution	AIC	BIC	A-BIC	Entropy	BLRT	Profile: *n*	LCP
1 profile	46,498.92	46,731.06	46,559.64	n/a	n/a	1. *n* = 544	
2 profiles	43,016.50	43,369.01	43,108.71	0.92	*p* < 0.001	1. *n* = 238	0.97
						2. *n* = 306	0.98
3 profiles	41,741.40	42,214.28	41,865.10	0.93	*p* < 0.001	1. *n* = 115	0.98
						2. *n* = 278	0.97
						3. *n* = 151	0.97
4 profiles	41,358.43	41,951.69	41,513.62	0.90	*p* < 0.001	1. *n* = 99	0.97
						2. *n* = 183	0.93
						3. *n* = 182	0.93
						4. *n* = 80	0.95
**5 profiles**	**41,026.03**	**41,739.66**	**41,212.71**	**0.90**	***p* < 0.001**	**1. *n* = 60**	**0.94**
						**2. *n* = 58**	**0.95**
						**3. *n* = 185**	**0.93**
						**4. *n* = 161**	**0.94**
						**5. *n* = 80**	**0.96**
6 profiles	40,741.31	41,575.31	40,959.45	0.92	*p* < 0.001	1. *n* = 32	0.94
						2. *n* = 75	0.96
						3. *n* = 159	0.95
						4. *n* = 174	0.93
						5. *n* = 84	0.94
						6. *n* = 20	0.95

*AIC = Akaike Information Criterion; BIC = Bayesian Information Criterion; A-BIC = Sample Size Adjusted BIC; BLRT = Bootstrap Likelihood Ratio Test; n/a = not available; n = number of participants; LCP = latent class probability. Bold font indicates selected model.*

#### Description of Identified Profiles

**FIGURE 3:**
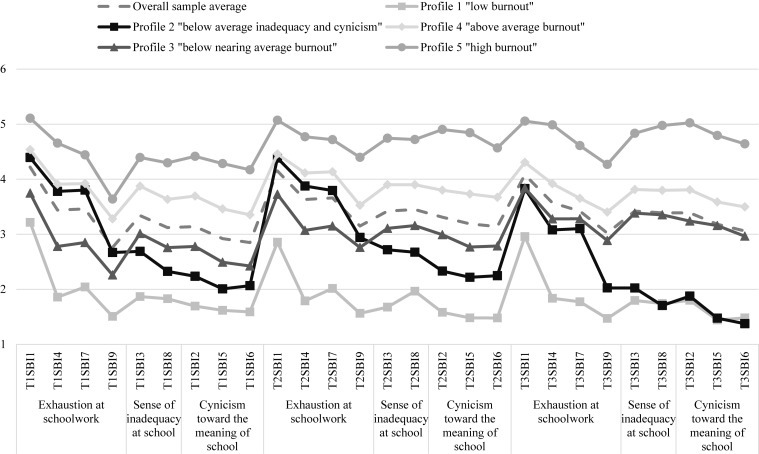
Study 2 conditional response means for a five-profile model.

The conditional response means for each indicator of school burnout assessed at T1, T2, and T3 are presented in Figure 3 and were used to interpret each class. Participants in profile 1 (approximately 11% of the overall sample) reported the lowest levels of school burnout across all three time points and were labeled *low burnout.* Those comprising profile 2 (10%) reported significantly lower-than-average levels of sense of inadequacy at school and cynicism toward the meaning of school but mean levels of exhaustion across all three time points, and therefore were labeled *below-average inadequacy and cynicism*. Profile 3 (34%) was labeled *below-nearing-average burnout* because it contained participants with below-average levels of burnout at T1 and T2 that approached average levels of burnout at T3. Participants in profile 4 (30%) reported above-average levels of burnout across all three time points and were labeled *above-average burnout.* Lastly, profile 5 (15%) was labeled *high burnout* because it contained participants who reported the highest levels of burnout across all three time points.

#### Differences in Sleep Disturbance as a Function of Profile Membership

**FIGURE 4:**
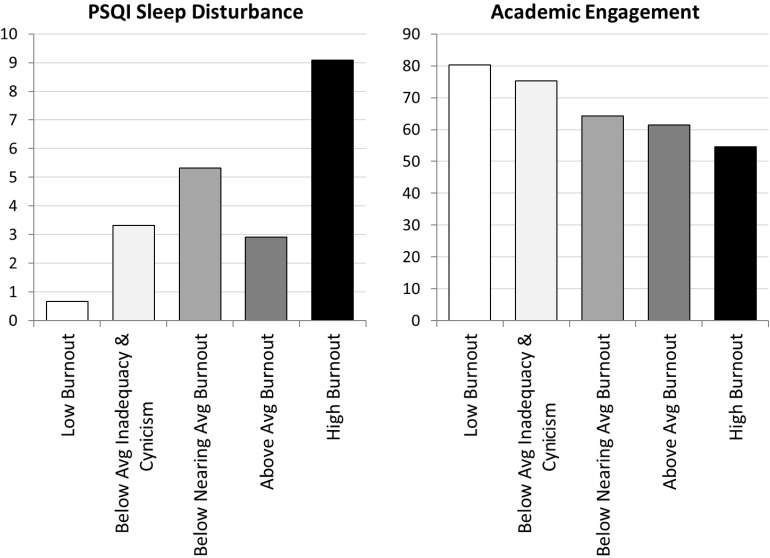
Study 2 differences on sleep disturbances and academic engagement across burnout profiles. PSQI, Pittsburgh Sleep Quality Index.

Results from the Wald chi-square significance tests indicated that levels of sleep significantly varied across profiles (Figure 4). Table 4 shows *post hoc* comparisons in covariates between profiles. Participants in the *low burnout* profile reported the lowest level of sleep problems (*M* = 0.67), whereas those comprising the *high burnout* profile were significantly higher on this measure and recorded the highest levels of sleep problems during the last data assessment wave (*M* = 9.09). There were no significant differences in sleep scores between the *below-average inadequacy and cynicism* and *above-average burnout* profiles.

**TABLE 4:** Profile differences in sleep and school engagement at time 3 in study 2 (*N* = 544).

Outcome *M* (SE)	Profile 1: “low burnout”	Profile 2: “below-average inadequacy and cynicism”	Profile 3: “below-nearing- average burnout”	Profile 4: “above- average burnout”	Profile 5: “high burnout”
Sleep disturbances	0.67 (0.58)^a,b,c,d^	3.31 (0.42)^a,e,f^	5.32 (0.45)^b,e,g,i^	2.91 (0.71)^c,g,h^	9.09 (0.73)^d,f,h,i^
Academic engagement	80.82 (1.58)^a,b,c,d^	75.20 (1.66)^a,e,f,g^	64.33 (1.20)^b,e,h^	61.42 (1.15)^c,f,i^	54.57 (1.78)^d,g,h,i^

*M = Mean; SE = Standard error. a, b, c, d = Matching subscripts denote significant post-hoc difference.*

#### Differences in Academic Engagement as a Function of Profile Membership

Results also indicated that levels of academic engagement significantly varied across profiles (Figure 4). Those comprising the *low burnout* profile recorded the highest levels of engagement (*M* = 80.02), followed by participants in the *below-average inadequacy and cynicism* profile (*M* = 75.20), *below-nearing-average burnout* (*M* = 64.33), and *above-average burnout* (*M* = 61.42), and those in the *high burnout* profile reported the lowest levels of engagement (*M* = 54.57). There were no significant differences in academic engagement scores between the *below-nearing-average burnout* and *above-average burnout* profiles.

Study findings from the RMLPA suggested a five-profile solution. With the inclusion of profile 2, *below-average inadequacy and cynicism*, this study produced a one-profile class increase from Study 1. However, it should be noted that this was the smallest class produced by the profile analysis as it comprises only 10% of the sample. Interestingly, in regard to trajectory, the analysis demonstrated fairly consistent membership trajectories across time—with approximately 66% of the study sample having a stable trajectory. The finding that the majority of the sample displayed a stable school burnout trajectory over time is consistent with the European school burnout literature (Parker and Salmela-Aro, 2011; Salmela-Aro and Upadaya, 2014). For example, Salmela-Aro and Upadaya (2014) demonstrated in their first study that about 60% of the sample showed a relatively stable burnout trajectory. However, it should be stated that about a third of the students (34%) in the current study showed a slowly increasing trajectory from below-average to near-average burnout. The current profile identification findings are also somewhat consistent with Study 1 in Salmela-Aro and Upadaya (2014), demonstrating a four-latent-group solution (60% of the adolescents showed a low and stable level of school burnout, 29% increasing burnout, 3% strongly increasing burnout, and 8% high-decreasing school burnout), but not Study 2, where only two groups were identified (4% moderate and slightly decreasing and 6% high-increasing).

While this study was designed to provide a description of potential trajectory stability, future research may seek to test factors that help predict stability or change, such as that of experiencing educational stage transitions as examined in Salmela-Aro and Upadaya (2014). For the current data, it may be that the subgroup experiencing a slowly increasing trajectory from below-average to near-average burnout may be nearing or undergoing stressful educational transitions. However, this growth is probably more than an issue of simply transitioning into another year of school, as mean values of school burnout have not been shown to differ by undergraduate year in school (see May et al., 2014a, b).

In relation to covariates, our hypotheses were supported. Both sleep disturbance and academic engagement displayed patterns largely consistent with variable-oriented statistical approaches: the lowest school burnout profile reported the lowest level of sleep problems and greatest amount of academic engagement, whereas the highest school burnout profile reported the highest levels of sleep problems and least amount of academic engagement. However, highlighting the importance of person-oriented analysis, it could be argued that for sleep disturbance, a slightly more interesting pattern appeared, with one group producing an unexpected mean (profile 4: *above-average burnout*). While this pattern needs to be replicated, this finding would have been obscured by traditional correlational analyses. Overall, in addition to reporting novel findings using a United States undergraduate student sample, this study adds to the school burnout literature in that relatively little is known regarding sleep quality and school burnout in any undergraduate student population.

In evaluating the profile findings from Studies 1 and 2, these person-oriented school burnout results parallel those yielded by variable-oriented statistical approaches using the SBI. Furthermore, the additional covariate analyses (GPA, depression, anxiety, sleep, and engagement) largely mirror the school burnout findings of research in European populations. However, given the potential sample and response pattern differences between this American school burnout research and that conducted in other countries, it may be helpful to continue to refine and develop the SBI. Therefore, to supplement the person-oriented SBI analyses of Studies 1 and 2, we now provide an IRT analysis of the SBI.

## Study 3

Item response theory (Hambleton et al., 1991) refers to a conglomeration of statistical models and techniques (e.g., latent distribution theory, item characteristic curve, item characteristic function, differential item functioning) that have been used to augment the limitations of classical test theory (CTT) approaches to measurement scale development and evaluation. CTT approaches to measurement rely mainly on correlational techniques like Cronbach’s alpha coefficients, exploratory factor analysis (EFA), and confirmatory factor analysis (CFA). Although CTT approaches have advantages (e.g., require smaller sample sizes, weaker assumptions) and can be effective at creating internally consistent scales, researchers are using IRT approaches to develop psychometrically optimized scales by increasing precision and minimizing measurement error (Foster et al., 2017; Mahmud, 2017).

Item response theory accomplishes this optimization by evaluating the latent trait that determines how individuals respond to test items. Specifically, IRT estimates latent scores (θ) for each participant on the construct being examined. IRT then evaluates the response curves of each item to determine if participants with higher θ scores select higher response choices and participants with lower θ scores select lower response choices. If this is then true for an item, it is considered effective and informative for assessing θ. Through this process, IRT analyses provide estimates of the discriminating information that each item can provide a measurement scale. Although IRT requires stronger assumptions and considerably larger sample sizes, item parameters yielded by IRT analysis are largely subpopulation-invariant, helping to produce test items and measurement scales that can function consistently in a wide range of future samples. Thus, item parameter invariance is a major advantage of IRT over CTT as it allows researchers to generalize how items work across populations.

Use of IRT techniques to supplement traditional measurement analyses of burnout (e.g., EFA, CFA) have been minimal given the large amount of burnout research. Only a few evaluations have been conducted, and these are limited to the Oldenburg Burnout Inventory (see Gustavsson et al., 2010; Reis et al., 2015) and variants of the Maslach Burnout Inventory [see Gonzalez-Roma et al., 2006 analysis of the Maslach Burnout Inventory-General Survey (MBI-GS) and Denton et al., 2013 analysis of the Maslach Burnout Inventory-Educators Survey (MBI-ES)]. Furthermore, there have been no published IRT-based analyses of the SBI and only limited information regarding its factor structure provided via traditional measurement analyses. Arguably Salmela-Aro et al. (2009) conducted the most comprehensive evaluation of the factor structure of school burnout, concluding via factor analysis that a model where three first-order burnout factors are explained by a second-order factor measuring overall school burnout fit the data the best. Thus, as evaluations of the factor structure of the SBI are limited, this study sought to evaluate the SBI in an American undergraduate sample using factor analysis and IRT.

### Methods

#### Participants

Undergraduate students who completed at least one full academic semester were eligible for study participation (*n* = 2,364, 86% females, *M*_*age*_ = 20.11 years, SD = 1.95). Sample demographics include: 68% Caucasian, 11% African American, 4% Asian, 15% Hispanic, and 2% endorsing either biracial or non-disclosed ethnicity, with 16% freshmen, 35% sophomores, 31% juniors, and 18% seniors. This sampling was conducted independently from Studies 1 and 2; thus, there was no overlap in participant data.

#### Measures

##### Anxiety symptoms

As in Study 1, anxiety was measured using the 20-item State-Trait Anxiety Inventory (STAI; Spielberger et al., 1970). Internal consistency for the present sample was α = 0.91.

##### Depressive symptoms

As in Studies 1 and 2, depressive symptomology was assessed via the 10-item CES-D (Radloff, 1977; Santor and Coyne, 1997). Internal consistency for the present sample was α = 0.92.

##### School burnout

As in Studies 1 and 2, school burnout was measured using the nine-item SBI (Salmela-Aro et al., 2009).

##### Perceived stress

Perception of stress over the past month was assessed using the 10-item Perceived Stress Scale (PSS-10; Cohen et al., 1983). The PSS-10 has respondents rate items (e.g., “In the last month, how often have you been upset because of something that happened unexpectedly?” and “In the last month, how often have you felt that you were unable to control the important things in your life?”) on a scale from 0 (*never*) to 4 (*very often*) or from 1 (*completely disagree*) to 6 (*completely agree*). Internal consistency for the present sample was α = 0.85.

#### Procedure

Data collection from all eligible participants was completed via online survey questionnaires over the course of two academic years (six semesters). Questionnaires contained demographic questions and the measurement instruments described. All participants were recruited from undergraduate classrooms at a large southern university in the United States as an option for voluntary class credit. Extra credit was generally less than 1% of the final grade. Data were collected between weeks 3 and 5 of each academic semester. All participants gave their written consent prior to study participation, and approval was obtained from the institutional review board before any data were collected.

### Results and Discussion

Item response theory assumes the items being examined assess a single construct (i.e., that they are unidimensional). An EFA on the nine items of the SBI yielded a dominant first factor with an eigenvalue (4.64) that accounted for 52% of the variance and was over four times larger than the second eigenvalue (1.13), suggesting that the items could reasonably be considered unidimensional. An IRT analysis was therefore performed on the SBI items to identify the items most effective at discriminating burnout between students. To perform this IRT analysis, graded response model (GRM; Samejima, 1997) parameters for the items within each set were estimated with Multilog 7.0 (Thissen et al., 2002) using marginal maximum likelihood estimation. GRMs are well suited for analyzing polytomous data such as Likert-based SBI response items.

**FIGURE 5:**
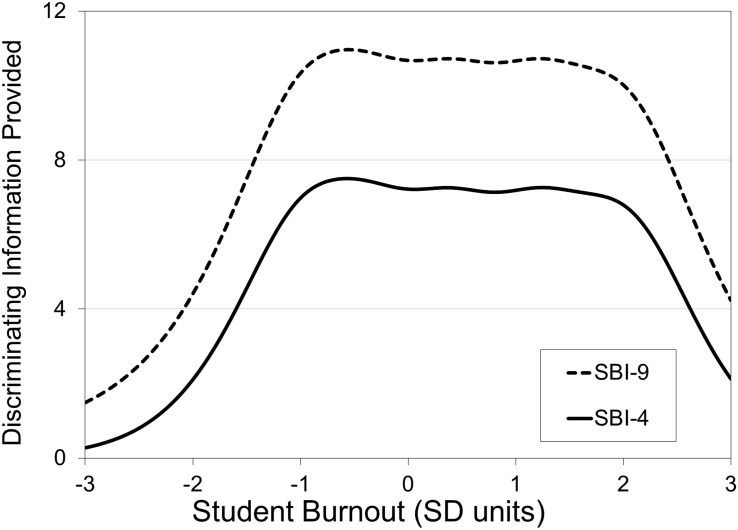
Test information curves for the nine-item and four-item Student Burnout Inventory (SBI).

As described in the introduction, IRT conceptualizes the information provided by an item as that item’s ability to discriminate between individuals on the construct being measured (termed θ in IRT equations). Thus, an item is considered to be more informative if subjects lower on θ select lower answer choices and subjects higher on θ select higher answers. IRT specifically evaluates how the distributions of the responses for each item map onto the latent θ estimates across all subjects (generating item response curves represented by GRM item parameters) to create information profiles (termed item information curves or IICs at the item level and test information curves or TICs at the scale level; see Figure 5). Information curves reveal how much discriminating information items or scales provide at various levels of θ (ranging from 3 standard deviations below the mean to 3 standard deviations above the mean). Information curves therefore synthesize the item parameters estimated by the GRM to provide a graphic method of comparing the relative information provided for items and scales from the same analysis, with curves of greater height (more information) and greater width (spanning a greater range of q) identifying highly effective items and scales. Put simply, the greater the area under any information curve, the greater the discriminating information offered.

The IICs and the GRM item discrimination parameters were examined and identified four SBI items providing the largest amount of information across the widest range of student burnout, thereby leading to identification of the SBI-4. These four items are items 2, 3, 5, and 6 from Salmela-Aro et al. (2009): “I feel a lack of motivation in my schoolwork and often think of giving up,” “I often have feelings of inadequacy in my schoolwork,” “I feel that I am losing interest in my schoolwork,” and “I’m continually wondering whether my schoolwork has any meaning,” respectively. It is important to note that none of these items are represented in the exhaustion subscale, as items 2, 5, and 6 come from the cynicism subscale and item 3 from the inadequacy subscale.

**FIGURE 6:**
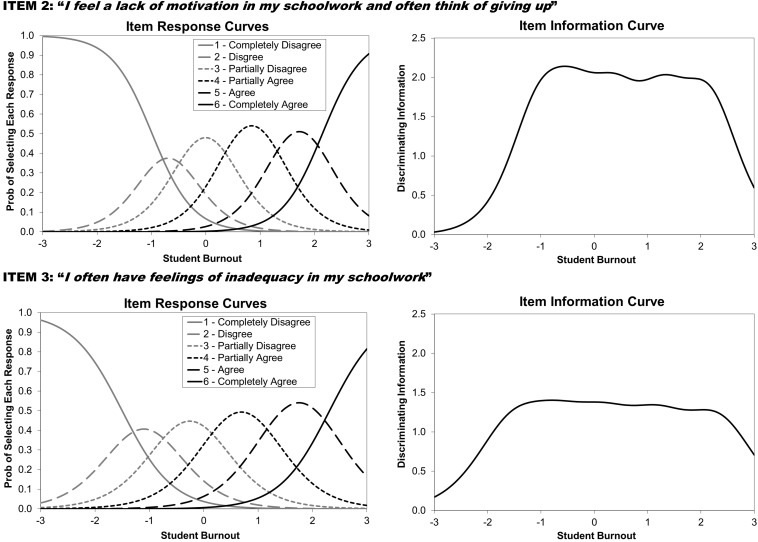
Item response theory (IRT)–generated item curves for school burnout items 2 and 3.

**FIGURE 7:**
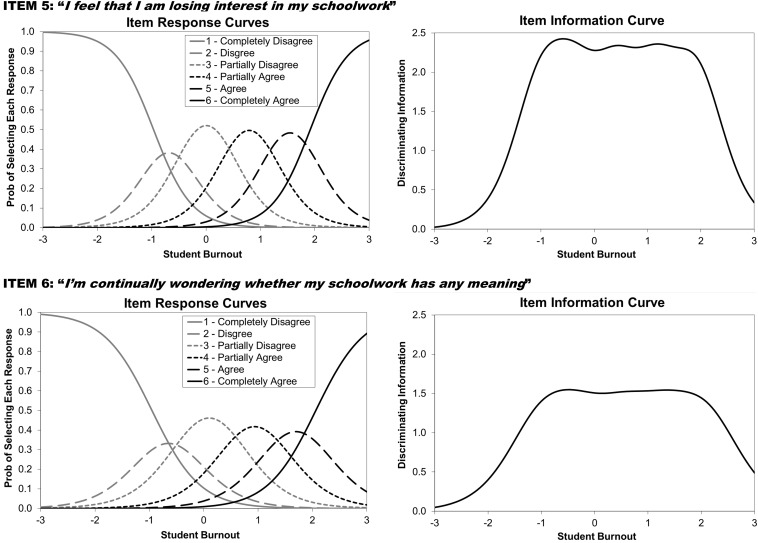
IRT-generated item curves for school burnout items 5 and 6.

Figure 5 shows the TICs for the original nine-item SBI as well as the new SBI-4. As revealed by the information curves, although the SBI-4 is less than half the length of the original scale, it offers high levels of information for assessing burnout. Demonstrating the high degree of information the SBI-4 carries in comparison to the nine-item SBI, the SBI-4 and the nine-item SBI correlated at *r* = 0.92. Furthermore, Pearson correlations between the measurement scales and the SBI-4 and the nine-item SBI produced highly similar patterns: CES-D (SBI-4 *r* = 0.49, SBI-9 *r* = 0.52), STAI (SBI-4 *r* = 0.45, SBI-9 *r* = 0.49), and PSS-10 (SBI-4 *r* = 0.51, SBI-9 *r* = 0.60). IICs for SBI items can be found in Figure 6 (items 2 and 3) and Figure 7 (items 5 and 6).

## General Discussion

Over three independent studies, this research evaluated school burnout as measured by the SBI through latent profile (person-oriented) and IRT analyses in American undergraduate students. Studies 1 and 2 identified mutually exclusive school burnout subgroups via LPA. These subgroups were linked to meaningful indicators of both academic success (GPA and academic engagement) and health (depression, anxiety, and sleep quality) and suggested that the information gained from the person-oriented burnout profiles largely parallels that gained from traditional variable-oriented approaches. Thus, latent prolife approaches revealed that respondents can be (generally) ordered reliably according to their symptom severity (i.e., the score alone likely provides enough information about between-person differences). Findings also suggest that school burnout follows a stable trajectory over time for the majority of students. Supplementing the profile analyses, IRT analysis provided a novel, more concise 4-item school burnout measure (SBI-4) that provides reasonably high levels of information for assessing school burnout. This research therefore produces a novel contribution to the school burnout literature by providing the initial evaluation of person-oriented and IRT-based analytic evaluations of burnout in American students.

Supplementing the more commonly found variable-oriented statistical approaches, the person-oriented analyses conducted in this research were able to identify an atypical school burnout typology. Whereas the LPA in Study 1 clustered subscale burnout profiles largely equivocally (mean values of exhaustion, cynicism, and inadequacy were approximately equivalent), the RMLPA in Study 2 identified an atypical school burnout profile where cynicism and inadequacy scores were discrepant from exhaustion scores. While small in prevalence in the sample, future research may find it fruitful to (1) identify covariates predictive of this divergence and (2) identify negative outcomes that may be more closely aligned with this profile than with the other clusters. Also of potential interest to future research may be latent transition analyses (LTAs). LTA has the ability to evaluate whether individuals change or switch their school burnout profile membership over time. The lack of modeling membership transition over time is a limitation of the current analyses.

The development of a shorter, more concise measure of the SBI (SBI-4) was a welcome outcome of the current research. This condensed measure is more time efficient and may be beneficial in time-sensitive data collection studies such as those conduced in medical-based research, diary-based studies, or larger, national or multi-site research projects. Advantageously, the SBI-4 is not only more time effective, but it does not suffer from information loss in comparison to the full SBI, as demonstrated by the IRT analysis. How the SBI-4 compares to other measures of school burnout such as the Maslach Burnout Inventory—Student Survey or the Oldenburg Burnout Inventory is a task for continued research.

The current IRT analysis may also have produced the added benefit of sparking continued interest in gaining a deeper and richer understating of how individuals respond to items and view the burnout construct, especially in student samples. This is highlighted by the fact that the SBI-4 contains no exhaustion items yet still carries considerable measurement information. Given these findings and noting that the graded response model provides information regarding how well an item differentiates between similar people, it may be that while the “amount” of exhaustion coincides with the construct of burnout (and is a necessary prerequisite of burnout), cynicism and inadequacy items better predict individual differences in burnout-covariate associations. Regardless, debates regarding the conceptualization of burnout (especially with emphasis being placed largely on the exhaustion dimension) and its measurement are far from concluded and appear more important than ever.

Notwithstanding the strength of this research, important limitations are worth addressing. One limitation is that the samples were predominantly female, thereby limiting the discovery of potential gender differences. Considerable burnout research suggests that females report greater levels of burnout in comparison to their male counterparts; thus, the current findings may be overestimating these psychosocial effect sizes. However, it should be noted that in regard to physiological functioning, prior research indicates that school burnout is associated with cardiovascular risk similarly in both male and female undergraduates (May et al., 2014a, b). Another limitation is the use of self-reported GPA. As an alternative strategy, grades might have been collected from the university registrar, potentially decreasing the influence of self-report bias. However, investigations consistently demonstrate that self-reported grades in undergraduates correlate greatly (*r* > 0.80) with actual grades (Kuncel et al., 2005).

Importantly, it should be emphasized that the current research only focused on undergraduates assessed cross-sectionally or in a relatively short time frame. The three consecutive semesters used in this research may even be too short of a window to fully capture the developmental profile of burnout. It may be advantageous for future research to evaluate the burnout process over multiple years and in multiple samples, evaluating the same hypotheses tested here in more diverse populations ranging from primary to tertiary school populations (e.g., school-aged children to adults enrolled in medical school programs, Ph.D. graduate programs, and law school programs). Finally, in Study 3, only a subset of possible IRT analyses were conducted. As noted regarding the potential addition of LTAs, additional IRT-based analyses may lead to more confident conclusions regarding subpopulation invariance (for example, contrasting or supplementing IRT differential functioning analyses with IRT likelihood ratio tests).

In summary, the current findings show that school burnout manifests itself in unique clusters of symptoms, which are largely stable over time. Covariate analyses demonstrated differences across profiles, but the patterns were similar to variable-oriented statistical approaches. This research also produced a novel, more concise and time-efficient measure of school burnout, the SBI-4. We believe the current research adds greatly to the understanding of burnout, especially burnout within academic settings (i.e., school burnout). This is especially true given the scarcity of person-oriented and IRT-based approaches in the investigation of school burnout and the absence of research on these topics in United States undergraduate student samples.

## Data Availability Statement

The datasets generated for this study are available on request to the corresponding author.

## Ethics Statement

The studies involving human participants were reviewed and approved by the Office for Human Subjects Protection – Florida State University. The patients/participants provided their written informed consent to participate in this study.

## Author Contributions

RM contributed to the data collection and manuscript writing. PR conducted the statistical analyses in Studies 1 and 2, and provided interpretation. RR conducted the statistical analysis in Study 3 and provided interpretation. FF contributed to the manuscript writing.

## Conflict of Interest

The authors declare that the research was conducted in the absence of any commercial or financial relationships that could be construed as a potential conflict of interest.
